# Efficacy of a combination of 10% imidacloprid and 1% moxidectin against *Caparinia tripilis* in African pygmy hedgehog (*Atelerix albiventris*)

**DOI:** 10.1186/1756-3305-5-158

**Published:** 2012-08-07

**Authors:** Kyu-Rim Kim, Kyu-Sung Ahn, Dae-Sung Oh, Sung-Shik Shin

**Affiliations:** 1 Department of Parasitology, College of Veterinary Medicine, Chonnam National University, Gwangju, 500-757, Korea; 2 Biotherapy Human Resources Center, College of Veterinary Medicine, Chonnam National University, Gwangju, 500-757, Korea

**Keywords:** African pygmy hedgehog, *Atelerix albiventris*, *Caparinia tripilis*, moxidectin, imidacloprid

## Abstract

**Background:**

The efficacy and safety of a combination formulation of 10% imidacloprid + 1.0% moxidectin spot-on (Advocate^®^ for Cats, Bayer Animal Health GmbH, Leverkusen, Germany) was tested in 40 African pygmy hedgehogs (*Atelerix albiventris*) naturally infested with *Caparinia tripilis*.

**Methods:**

The optimal dosage level of the combination for hedgehogs was determined by assigning 20 hedgehogs into three treatment groups (0.1, 0.4 and 1.6 ml/Kg b.w.), and one untreated control group of 5 hedgehogs each. Twenty naturally infested hedgehogs were then randomly assigned to either treatment or control group with 10 animals each, and the number of live mites was counted from 13 body regions on day 0, 3, 9, 16, and 30 after single treatment at the dosage level of 0.1 ml/Kg.

**Results:**

Before the chemotherapy, the highest density of mite was observed in external ear canals followed by the dorsal and the lowest in the ventral regions of the body surface. The dosage level of 0.1 ml/Kg, which corresponded to the recommended dosage level for cats, containing 10 mg imidacloprid and 1 mg moxidectin was also the optimal dosage level for hedgehogs. No hedgehogs in the treatment group showed live mites from day 3 post treatment. Side effects such as ataxia, depression, nausea, and weight fluctuation were not observed during the whole period of study.

**Conclusions:**

This report suggests that a combination formulation of 0.1 ml/Kg of 10% imidacloprid + 1% moxidectin spot-on for cats is also useful for the control of *Caparinia tripilis* infestation in hedgehogs.

## Background

*Caparinia tripilis* (Michael, 1889), first reported in *Erinaceus europaeus* from England [1], is one of the most important causes of skin disease in hedgehogs [2]. The ears of infested animals become scaly and the skin of the affected parts thickens. The mite causes annoyance to their hosts, which shows pruritic symptoms as they often sit on their haunches and attempt to scratch infested areas with their hind claws. In advanced cases, dry crusts form on the surface of the skin, which thickens, becomes folded, and may even crack. Bleeding can occur when the lesions become worse, spines may fall out, and affected hedgehogs eventually become so incapacitated that they cannot roll up properly [3]. The life cycle of *C. tripilis* includes egg, larva, protonymph, deutonymph (none of which shows sexual dimorphism) and either an adult male or an adult female [3]. Observations suggest that the complete life cycle encompasses about three weeks [3]. By means of genital suckers, an adult male and a pubescent female (deutonymph) form an attachment pair, which is more or less permanent up until the time of emergence of the adult female from the deutonymphal exuviae. Severe infestation may be found in young animals as early as the age of 4 months and heavily infested animals may die [3]. Infested hedgehogs, therefore, must be isolated and treated.

Treatments in hedgehogs have included organophosphate bath, ivermectin injection, and amitraz spray [4,5]. After the treatment with organophosphate bath (Paramite^TM^, Vet-Kem Co., Dallas, Texas), skin lesions were improved and the condition had not recurred [4]. However, organophosphate insecticide toxicity is the leading cause of major morbidity and death in the insecticide class. The clinical syndrome of organophosphate toxicity varies widely, ranging from the classic cholinergic syndrome to flaccid paralysis and even intractable seizures [6]. While ivermectin (Eqvalan^®^, Merck & Co., Rahway, New Jersey) administered intramuscularly at 21-day intervals reduced clinical disease caused by *Caparinia* infestation in *A. albiventris*, it failed to eliminate the mites. On the other hand, two treatments with a 0.03% rinse of amitraz (Mitaban^®^, Upjohn Co., Kalamazoo, Michigan) 7 days apart cleared the infestation [5]. An application of 1% permethrin has also been shown to be effective [7].

Moxidectin, a macrocyclic lactone disaccharide, is a potent, broad-spectrum endectocide with activity against a wide range of nematodes, insects, and mites. First used commercially in Argentina as an injectable formulation for cattle in 1989, it has been widely used as a broad-spectrum antiparasitic remedy for a variety of mammalian species including food-producing and companion animals [8]. It has been reported that moxidectin is 100 times more lipophilic than ivermectin and that the concentration of moxidectin in fat tissue was 90-fold higher than that detected in plasma 28 days following treatment in cattle [9,10]. The high liphophilic nature of moxidectin may be particularly important as a potent miticide against those parasitic mites that live on the surface of animal skin with sebaceous glands. Recent experimental studies confirmed the efficacy of topical imidacloprid + moxidectin against otoacariosis caused by *Otodectes cynotes* in dogs [11] and cats [12]. The imidacloprid + moxidectin formulation were also highly efficacious against the KS1 *Ctenocephalides felis*, flea strain infesting cats [13]. With the ease of a spot-on medication, the formulation could also be used for parasitic control in exotic pet animals, and studies on ear mite infection caused by *O. cynotes* in ferrets resulted in successful elimination of the parasite [14]. However, the efficacy and side effects against mange infestation in hedgehogs have not been studied yet. The objective of the present investigation is to evaluate the efficacy and safety of imidacloprid 10% + moxidectin 1% spot-on against naturally infested *Caparinia tripilis* in hedgehogs.

## Methods

### Study animals

In November 2010, forty African pygmy hedgehogs (*Atelerix albiventris,* 13 male and 27 female, mean ± SD body weight, 305.6 ± 111.2 g) naturally infested with *Caparinia tripilis* were obtained from a local pet shop in Gwangju, Korea. A brief description of the hedgehogs' sex, body weight, and coloring was recorded. None of the hedgehogs had been treated with ectoparasiticides in the last 6 months before the experiment. The hedgehogs were kept in cages individually during the experiment. All animals were housed in the same room at 25.0 ± 2.0°C and 80% relative humidity, and were given regular commercial hedgehog food throughout the experimental period. The Institutional Animal Care and Use Committee at Chonnam National University approved the protocols used in this study, and the animals were cared for in accordance with the Guidelines for Animal Experiments.

For parasitological identification, skin scraping samples from severely affected animals were collected and preserved in 70% methanol. Mites were mounted on slides using Downs’ PVA solution for several days until the internal organs became transparent [15], and were identified by the morphology of adult males according to a key provided by Laurence [16].

### Drug

For the treatment against the mite infestation, a formulation product of 10% imidacloprid + 1% moxidectin spot-on for cats (Advocate^®^ for Cats, Bayer Animal Health GmbH, Leverkusen, Germany) was used.

### Study designs

#### Experiment 1. Determination of optimal dosage level for hedgehogs

Fifteen hedgehogs were randomly assigned to the treatment group and five to the untreated control group by a random treatment allocation plan generated with a Microsoft^®^ Excel macro (Microsoft Corporation, Redmond, WA, USA). The treatment group was divided into 3 subgroups of 5 hedgehogs each for 3 different dosage levels of the test drug. Treatment groups were administered once with different dosage levels of the test drug (0.1, 0.4 or 1.6 ml/Kg b.w.) on day 0 to the skin of the mid-dorsal region of each hedgehog using a pipette (Gilson Pipetman, p200, France) after the number of mites was counted. The control group received the same aliquot of 0.1% benzyl alcohol. The dosage level of 0.1 ml/Kg contained 10 mg imidacloprid and 1 mg moxidectin.

The main criterion for the drug efficacy was the absence of viable mites from the host animal at any developmental stage, including larva, nymph, and adult. The distinction of gender was not attempted because the nymph and adult stages were particularly difficult to differentiate on a fresh preparation unless the internal non-transparent organs and materials were cleared by mounting liquids such as PVA solution, which takes several days [15]. Mites were considered as live if they were motile and retained their body conformation. Enumeration of mites by conventional skin scraping and light microscopic examination was not used in this study because dead mites killed by the drug administration could erroneously be counted as live mites during light microscopic examination after KOH dissolution. Likewise, the ovicidal efficacy of the test drug was not evaluated because the viability of eggs could not be easily determined either by otoscopic or microscopic examination. Although no records for the genus *Caparinia* are available, eggs of the genera *Chorioptes* and *Otodectes* which are closely related to *Caparinia* in the family Psoroptidae, require an average of 4 days (range 72–107 hours) to hatch at 35°C and 80% relative humidity [17,18]. Therefore, successful control of eggs can be expected if larvicidal efficacy of the test drug is ensured to last at least for one week post treatment.

Before the administration of the drug on day 0, the number of live motile mites were counted from a total of 13 skin regions of each animal (6 dorsal [dorsal neck, left and right dorsal thoracic, left and right lumbar, and sacral regions], 5 ventral [left and right pectoral, left and right abdominal and inguinal regions] and external ears of both sides) using an otoscope (Piccolight^®^, KaWe, Berlin, Germany) for 30 seconds each. The number of mites on approximately 0.5 cm^2^ of body surface was counted for each region. On day 3, the number of live mites found in all twenty hedgehogs was counted by the same method as in Day 0. Every hedgehog was weighed with a scale to observe weight change at each examination.

#### Experiment 2. Efficacy evaluation

Ten naturally infected hedgehogs were randomly assigned to the treatment group and ten naturally infected hedgehogs to the untreated control group by a random treatment allocation plan generated with a Microsoft^®^ Excel macro (Microsoft Corporation, Redmond, WA, USA). Before the administration of the drug, the number of live mites found in all twenty hedgehogs was counted by the same methods as described in Experiment 1. Advocate^®^ for Cats (Bayer Animal Health GmbH, Leverkusen, Germany) at the dosage level of 0.1 ml/Kg was administered to the skin of the mid-dorsal part of hedgehogs in the treatment group as a single treatment on day 0. All animals were examined 5 times, on day 0, 3, 9, 16, and 30 during the experiment. The number of live mites found in 13 body regions was counted by the same method as described in Experiment 1. After all 13 body regions were examined by an otoscope, the viability of mites was double checked by collecting skin scraping samples from the posterior dorsal neck region of each hedgehog into a disposable polystyrene dish (IWAKI 150x20mm, Asahi Glass Co., Ltd., Japan) and examined under a stereoscope (Stemi 2000-C, Zeiss) for live mites. All animals were weighed with a scale on each examination. The efficacy of the drug in each treatment group for Day 3 and after was calculated as follows:

(1) $$\text{The percentage of efficacy}\;\left(\%\right)=\frac{y-x}{y}\times 100$$

x= number of hedgehogs observed with live mites

y = total number of hedgehogs in group

### Statistical analysis

A Kruskal-Wallis test was used to evaluate differences in the population density between body regions. Differences in body weight measured over time were evaluated through repeated-measure ANOVA models. The signification level was set at p ≤ 0.05. Statistical analysis was performed using IBM SPSS Statistics software version 19.0 (IBM Corporation, Armonk, New York, U.S.).

## Results

**Figure 1:**
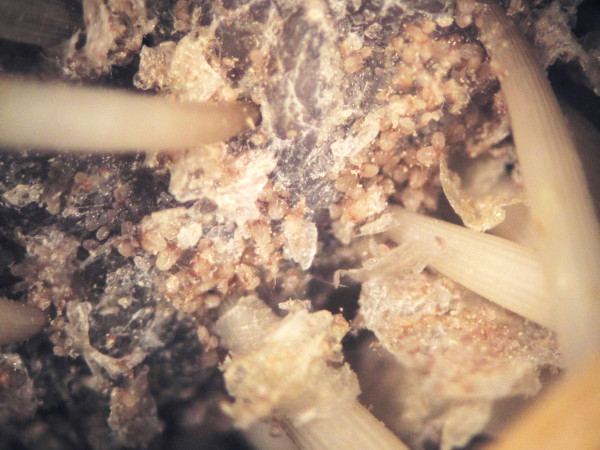
**Heavily-infested skin of a hedgehog (*Atelerix albiventris*) with* Caparinia tripilis.*** Numerous mites at various developmental stages including eggs are shown. Photo image taken from a dissecting microscope (Zeiss Stemi 2000-C).

**Figure 2:**
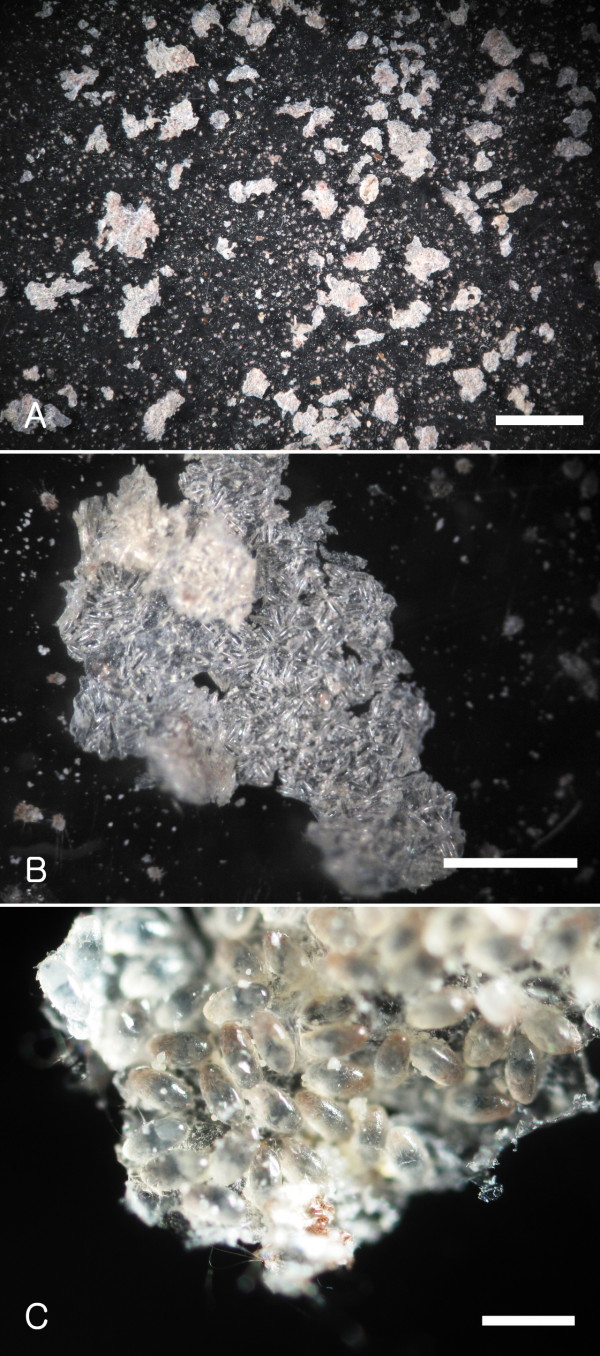
**Flakes of scale collected from a hedgehog heavily infested with* Caparinia tripilis.* A**, Low magnification of scales collected from an infested hedgehog. Scales were stored at room temperature for 4 weeks. Tiny dots of larvae hatched from eggs are shown. Bar = 5 mm. **B**, Photograph of an enlarged scale that is covered with mostly empty egg shells. Bar = 1 mm. **C**, Photomicrograph of an enlarged scale of a heavily-infested hedgehog showing numerous eggs. Bar = 200 μm.

**Figure 3:**
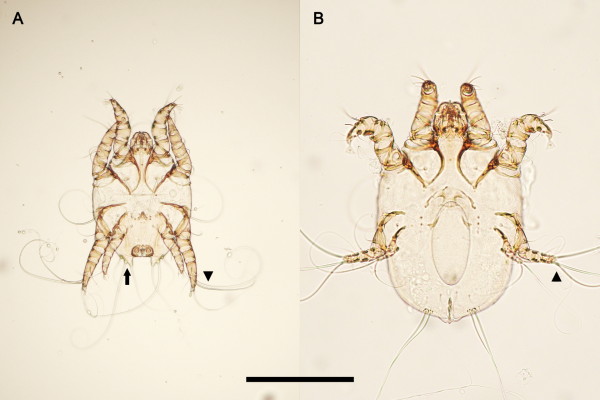
**Light micrographs of adult male and female* Caparinia tripilis*.A**, Ventral view of an adult male. A trilobate projection (black arrow) is present on each side of the posterior end of the abdomen, each lobe with a long seta. **B**, Ventral view of an ovigerous female (=adult female) containing an egg. Three long setae on the third pair of legs in both sexes are present (arrow head). Bar = 200 μm.

Figure 1 shows a stereoscope view of the affected skin of a naturally-infested hedgehog with numerous mites at various developmental stages. The mantle of severely populated regions of the skin was covered with scale and crust that were often carpeted with either live eggs or empty egg shells (Figure 2). Morphologically, pedicels of adult mites were short and unjointed. Tarsal caruncles were bell-shaped on all legs of males while they were absent on legs III and IV of females. Three long setae on the third pair of legs in both sexes were present. Adult males had posterior end of the abdomen with trilobate projection on each side and each lobe with a long seta (Figure 3). Based on these morphological characteristics, the mite was identified as *Caparinia tripilis*.

The distribution pattern of* C. tripilis* on the 13 body regions of 40 hedgehogs before chemotherapy is shown in Table 1. Among the total of 2,495 mites counted from the 13 body regions with mean number of 4.8 ± 5.9 mites (mean ± S.D.), the highest density of mite was observed in the left external ear canal (8.7 ± 8.5), and the lowest in the inguinal region (0.9 ± 1.8). In general, the greatest concentration was recognized in ears (8.5 ± 8.7), followed by dorsal (6.3 ± 5.5), and the lowest in the ventral regions (1.5 ± 2.4). The difference in the number of mites between ears, dorsal, and ventral regions was significant with the Kruskal-Wallis test (p<0.001).

**Table 1:** Distribution of* Caparinia tripilis* on 13 body regions of 40 African pygmy hedgehogs (*Atelerix albiventris*)

**Body Regions**		**Number of* Caparinia tripilis counted***		
		**Total**	**Mean**	**S.D.**
Ears	Left	340	8.7	8.5
	Right	323	8.3	8.9
	Subtotal	663	8.5	8.7
Ventral	Left pectoral	86	2.2	2.6
	Right pectoral	73	1.8	2.8
	Left abdominal	47	1.2	1.3
	Right abdominal	66	1.7	2.9
	Inguinal	37	0.9	1.8
	Subtotal	309	1.5	2.4
Dorsal	Dorsal neck	327	8.2	6.5
	Left dorsal thoracic	303	7.6	6.8
	Right dorsal thoracic	244	6.1	4.2
	Left lumbar	206	5.2	4.4
	Right lumbar	236	5.9	5.0
	Sacral	207	5.2	5.2
	Subtotal	1523	6.3	5.5
Total		2495	4.8	5.9

Before the administration of drug on day 0, the number of live motile mites (larvae, nymphs, and adults) was counted from 13 body regions of 40 hedgehogs using an otoscope (Piccolight^®^, KaWe, Berlin, Germany). Number of mites on approximately 0.5 cm^2^ of body surface was counted for each region.

### Experiment 1. Determination of proper dosage level

Results for the optimal dosage level of Advocate^®^ for Cats against *C. tripilis* infestation in hedgehogs are shown in Table 2. Most mites in the treatment group administered with a dosage level of 0.1 ml/Kg were found dead on day 3. Only one live mite was found in one of five animals at the dorsal neck region. Other treatment groups, which were administered at the dosage level of 0.4 and 1.6 ml/Kg, respectively, showed no live mites at any body region examined. On day 3 in the control group, on the other hand, the mean number of mites was maintained above 3.7 with the highest density found in the left ear and the lowest in the inguinal region. There were no side effects recognized even at the dosage level of 1.6 ml/Kg, which was equivalent to sixteen times the recommended dosage level for cats. A dosage level of 0.1 ml/Kg was therefore selected as the optimal dosage for hedgehogs for Experiment 2.

**Table 2:** Determination of optimal dosage level of a combination of 10% imidacloprid and 1% moxidectin spot on against *Caparinia tripilis* in African pygmy hedgehogs (*Atelerix albiventris*)

**Days post treatment**		**0**				**3**			
**Body regions**	**Dosage(ml/kg)**^**a**^	**0**	**0.1**	**0.4**	**1.6**	**0**	**0.1**	**0.4**	**1.6**
Ears	Left	11.6	8.4	15.5	15.8	7.0	0.0	0.0	0.0
	Right	8.8	11.2	12.3	12.2	13.0	0.0	0.0	0.0
	Mean	10.2	9.8	13.9	14.0	10.0	0.0	0.0	0.0
Ventral	Left pectoral	1.8	2.4	2.2	1.6	3.4	0.0	0.0	0.0
	Right pectoral	1.0	3.0	1.4	3.0	3.4	0.0	0.0	0.0
	Left abdominal	0.8	1.0	1.8	1.6	2.2	0.0	0.0	0.0
	Right abdominal	0.8	1.6	1.4	1.8	2.2	0.0	0.0	0.0
	Inguinal	0.2	0.2	0.6	2.4	0.4	0.0	0.0	0.0
	Mean	0.9	1.6	1.5	2.1	2.3	0.0	0.0	0.0
Dorsal	Dorsal neck	10.2	3.6	12.2	5.0	6.2	0.2	0.0	0.0
	Left dorsal thoracic	11.0	4.2	5.4	4.8	3.8	0.0	0.0	0.0
	Right dorsal thoracic	7.4	5.0	4.6	4.0	3.4	0.0	0.0	0.0
	Left lumbar	5.2	2.2	6.2	3.4	3.0	0.0	0.0	0.0
	Right lumbar	4.0	2.2	8.0	5.4	3.0	0.0	0.0	0.0
	Sacral	3.0	3.4	3.6	1.4	4.4	0.0	0.0	0.0
	Mean	6.8	3.4	6.7	4.0	4.0	0.0	0.0	0.0
Mean		5.1	3.7	5.8	4.8	4.3	0.0	0.0	0.0

^a^ Advocate^®^ for Cats (Bayer Animal Health GmbH, Leverkusen, Germany) containing 10% imidacloprid + 1% moxidectin was administered once on the mid-dorsal part of each hedgehog. Each number represents mean number of live mites observed from 5 hedgehogs using an otoscope (Piccolight^®^, KaWe, Berlin, Germany). Number of mites on approximately 0.5 cm^2^ of body surface was counted for each region.

### Experiment 2. Efficacy evaluation of drug

**Figure 4:**
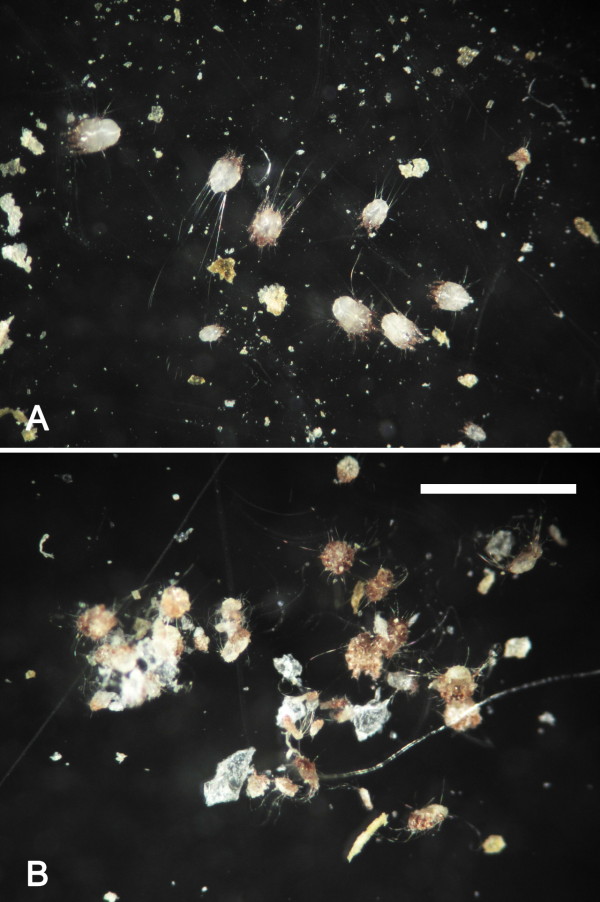
**Various stages of both live and dead mites of* Caparinia tripilis* collected from hedgehogs (*Atelerix albiventris*)**. **A**, Live mites collected from hedgehogs in the control group without any chemotherapy by skin scraping in the posterior dorsal neck region. Live mites can be easily distinguished from dead mites by their active motility and retention of their body conformation. **B**, Dead mites collected from hedgehogs 3 days after treatment with 0.1 ml/kg b.w. of 10% imidacloprid + 1% moxidectin (Advocate^®^ for Cats, Bayer Animal Health GmbH, Leverkusen, Germany). Bar = 1 mm.

A long-term miticidal efficacy of a formulation of 10% imidacloprid + 1% moxidectin against *C. tripilis* is shown in Table 3. While the average number of mites on the 13 body regions in the untreated control group maintained above 4.7 during the experimental period, hedgehogs in the treatment group showed no live mites from day 3 post treatment until the end of the experiment. The efficacy of 10% imidacloprid + 1% moxidectin on *C. tripilis* in hedgehogs was, therefore, 100% from day 3 to day 30. The efficacy of the drug was double confirmed by observing the viability of mites in skin scraping samples from the posterior dorsal neck region of each hedgehog under a stereoscope from which no live mites were observed from animals in the treatment group (Figure 4, data not shown).

**Table 3:** Efficacy of a combination formulation of 10% imidacloprid +1% moxidectin against naturally infested *Caparinia tripilis* in African pygmy hedgehogs (*Atelerix albiventris*)

**Groups**		**Control**					**Treatment**				
**Body regions**	**Days post treatment**^**a**^	**0**	**3**	**9**	**16**	**30**	**0**	**3**	**9**	**16**	**30**
Ears	Left	5.2	5.9	10.7	15.7	7.9	4.7	0.0	0.0	0.0	0.0
	Right	3.9	3.7	13.4	11.0	18.3	7.4	0.0	0.0	0.0	0.0
	Mean	4.6	4.8	12.1	13.4	13.1	6.1	0.0	0.0	0.0	0.0
Ventral	Left pectoral	2.8	0.6	1.8	0.8	0.5	1.8	0.0	0.0	0.0	0.0
	Right pectoral	1.3	0.5	1.6	0.6	1.2	1.8	0.0	0.0	0.0	0.0
	Left abdominal	0.8	0.6	1.4	1.0	1.1	1.3	0.0	0.0	0.0	0.0
	Right abdominal	0.9	0.6	1.0	0.5	1.0	2.9	0.0	0.0	0.0	0.0
	Inguinal	0.5	0.0	0.8	0.9	1.3	1.5	0.0	0.0	0.0	0.0
	Mean	1.3	0.5	1.3	0.8	1.0	1.9	0.0	0.0	0.0	0.0
Dorsal	Dorsal neck	9.1	13.8	8.3	16.0	7.4	8.1	0.0	0.0	0.0	0.0
	Left dorsal thoracic	5.7	15.3	7.3	9.6	5.6	11.9	0.0	0.0	0.0	0.0
	Right dorsal thoracic	6.4	12.2	7.6	9.0	5.8	7.5	0.0	0.0	0.0	0.0
	Left lumbar	6.5	9.6	8.2	7.0	5.5	5.6	0.0	0.0	0.0	0.0
	Right lumbar	7.2	10.8	8.2	6.9	5.1	6.6	0.0	0.0	0.0	0.0
	Sacral	10.5	11.4	8.9	10.7	9.0	4.5	0.0	0.0	0.0	0.0
	Mean	7.6	12.2	8.1	9.9	6.4	7.4	0.0	0.0	0.0	0.0
Mean		4.7	6.5	6.1	6.9	5.4	5.0	0.0	0.0	0.0	0.0
% Efficacy								100.0	100.0	100.0	100.0

^a^ Advocate^®^ for Cats (Bayer Animal Health GmbH, Leverkusen, Germany) containing 10% imidacloprid + 1% moxidectin was administered once on the mid-dorsal part of each hedgehog. For abbreviation, refer to Table 1. Each number represents mean number of live mites observed from 10 hedgehogs using an otoscope (Piccolight^®^, KaWe, Berlin, Germany). Number of mites on approximately 0.5 cm^2^ of body surface was counted for each region. The percentage of efficacy (%) = (y-x)/y*100 (x= number of hedgehogs observed with live mites in the test group for each observation date, y = total number of hedgehogs in the test group for each observation date).

The average weights of the control and treatment groups were 254 ± 88.2 g and 290 ± 164.1 g on day 0, respectively. On day 30, the average weight of the control group remained unchanged (254.0 ± 53.8 g) while that of the treatment group increased slightly (297.0 ± 137.1 g). The body weight change from day 0 to 30 was not statistically significant in both control (p=0.830) and treatment (p=0.198) groups. Side effects such as ataxia, depression, or nausea were not observed during the whole period of study in both control and treatment groups.

## Discussion

This study evaluated the efficacy and safety of 10% imidacloprid + 1% moxidectin (Advocate^®^ for Cats, Bayer Animal Health GmbH, Leverkusen, Germany) against naturally infested *Caparinia tripilis* in African pygmy hedgehogs. Though the drug we tested was primarily formulated for cat use, our results showed excellent efficacy and safety against the mange mite infestation in hedgehogs, too. The dosage level of 0.1 ml/Kg that was determined in Experiment 1 was also the recommended dosage for cats. Even on the third day post treatment, no hedgehogs in the treated group showed live mites in any body regions, which continued until the end of the experiment (30 days). In our study, side effects such as ataxia, depression, nausea, and weight fluctuation were not observed during the whole period of study. With the ease of use of a spot-on medication, especially for pet owners, the drug appears to be an ideal solution for the control of mange mite infestation in exotic pets such as hedgehogs.

Moxidectin, a macrocyclic lactone disaccharide, is about 100 times more lipophilic than ivermectin [10] and remains in the plasma much longer than ivermectin [19]. Once the drug enters the blood stream, it is stored in fat and slowly released, metabolized and excreted mainly via the feces but also appears on the skin where it kills surface-living mites. The miticidal efficacy of Advocate^®^ for Cats (10% imidacloprid + 1% moxidectin) against *C. tripilis* in this study may therefore be due to the activity of moxidectin because even those mites remotely located in the external ear canal were successfully killed. It has been reported that notoedric mange caused by *Notoedres cati* in a hedgehog was also treated with 0.3 mg/Kg moxidectin (Cydectin^®^ 1% injectable, Fort Dodge, IA, USA), administered subcutaneously, which was repeated after 10 days. Clinical signs improved significantly 10 days after the initial treatment [20]. Though the report did not record the number of mites before and after the treatment, moxidectin seemed to be effective for external parasite infestations in hedgehogs.

It appears that hedgehogs became popular as pets in the United States in the early 1990s when “Hazel the Hedgehog” turned up in an American comic strip in 1993 and “Sonic the Hedgehog” began to appear in the cartoon that bore his name [21]. The North American Hedgehog Association estimated that there were approximately 2,000 quality breeding animals in the United States in 1995 which remained rather stable in 2000 [21]. Although hedgehogs are legal to keep as pets in all of Canada and most areas of the United States of America, it is illegal to own them as pets in some US states and some Canadian municipalities, probably due to the ability of some hedgehog species to carry foot and mouth disease [22]. Only the African pygmy hedgehog can be legally kept in most European countries [21].

*Caparinia* infestion of hedgehog was first reported in England in 1889 by Michael who identified the mite as *Symbiotes(=Caparinia) tripilis*. The mite was then found in hedgehogs from Germany and New Zealand [2,23], and it has also been introduced to New Mexico, United States, through breeding colonies of African hedgehogs for sale as pets [7]. In Asia, an outbreak of *C. tripilis* infestation in a colony of African pygmy hedgehogs from Korea was reported in 2012 [24]. Human ringworm and salmonellosis cases acquired from African pygmy hedgehogs have also been reported [25,26], but *Caparinia tripilis* does not appear to be zoonotic [27].

## Conclusions

Although there has been a great increase in the population of hedgehogs as pets, limited studies on the control of ectoparasitic diseases of hedgehogs are available. This report suggests that a combination formulation of 0.1 ml/Kg of 10% imidacloprid + 1% moxidectin spot-on for cats is also useful for the control of *Caparinia tripilis* in hedgehogs.

## Competing interests

The authors declare that the conceptual design, the content or any other scientific aspect have not been influenced.

## Authors’ contributions

KRK and SSS planned the study design. KRK and KSA performed laboratory work. DSO analyzed the data. KRK drafted the manuscript. SSS provided substantial improvement of the manuscript and scientific supervision of the study. All authors approved the final version of the manuscript.
